# Impact of Middle East Respiratory Syndrome coronavirus (MERS**‐**CoV) on pregnancy and perinatal outcome

**DOI:** 10.1186/s12879-016-1437-y

**Published:** 2016-03-02

**Authors:** Haleema Alserehi, Ghassan Wali, Abeer Alshukairi, Basem Alraddadi

**Affiliations:** Division of Infectious Diseases, Department of Medicine, King Faisal Specialist Hospital and Research Centre, Jeddah, Kingdom of Saudi Arabia; King Faisal Specialist Hospital and Research Centre, P.O BOX 40047, Jeddah, 21499 Saudi Arabia

**Keywords:** MERS-COV, Pregnancy, ARDS, Perinatal outcome

## Abstract

**Background:**

Middle East Respiratory Syndrome coronavirus (MERS-CoV) is a viral respiratory disease. Most people infected with MERS-CoV develop severe acute respiratory illness. It was first reported in Saudi Arabia in 2012 and has since spread to several other countries. We report the clinical course of MERS-CoV infection in a pregnant woman who acquired the infection during the last trimester.

**Case presentation:**

The patient is a 33-year-old female working as a critical care nurse. She was 32 weeks pregnant when she presented with respiratory symptoms after direct contact with a MERS-COV patient. Although the patient was in respiratory failure, necessitated mechanical ventilation, and intensive care (ICU) admission, a healthy infant was delivered. The mother recovered. To the best of our knowledge, this is the first reported case of a laboratory-confirmed Middle East Respiratory Syndrome Coronavirus in a pregnant woman.

**Conclusions:**

Middle East Respiratory Syndrome coronavirus (MERS-CoV) known to cause severe acute respiratory illness associated with a high risk of mortality Various factors may have contributed to the successful outcome of this patient such as young age, presentation during the last stages of pregnancy, and possible differences in immune response.

## Background

Middle East Respiratory Syndrome coronavirus (MERS-CoV) is a novel coronavirus known to cause severe acute respiratory illness associated with a high risk of mortality. As of August 17 2015, 1432 laboratory-confirmed cases of infection with MERS-CoV, including at least 507 deaths, have been confirmed worldwide [1]. In pregnant women, the risk of viral pneumonia is significantly higher than for the rest of the population according to data collected from the previous 1957–1958 pandemics, and the H1N1 influenza pandemic of 2009 [2, 3]. Pregnant women with severe acute respiratory syndrome (SARS) appear to have a worse clinical outcome and a higher mortality rate compared to non-gravid women [4, 5]. Rates of maternal mortality, stillbirth, spontaneous abortion, and preterm delivery have all been elevated in viral pneumonia such as influenza-A, virus subtype H1N1, and SARS. While there are no clinical or serologic reports suggesting transmission of SARS coronavirus to the fetus, vertical transmission has been reported for H1N1 and Respiratory Syncytial Virus (RSV) [4, 6]. Data on the effects of MERS-CoV on pregnancy are limited; two cases of MERS-CoV in pregnancy have been reported to this day. The first report involved a stillbirth at 5 months of gestation in a woman with MERS-CoV infection in Jordan [7]. The other involved a woman in the United Arab Emirates with MERS-CoV infection during the 3rd trimester who died after giving birth to a healthy baby with no evidence of MERS-CoV infection [8]. We report the clinical course of MERS-CoV infection in a pregnant woman who acquired the infection during the last trimester of pregnancy during a large hospital outbreak.

## Case presentation

The patient was a 33-year-old female working as a critical care nurse, with a prior history of hypothyroidism and primary infertility. She had undergone successful in vitro fertilization prior to presentation. She had been exposed to a patient with a respiratory illness, later identified as MERS-CoV infection, and was using standard contact and droplet precautions while under her care. Three days after exposure, she began to exhibit a dry cough and fever prompting her to visit a family medical clinic, where she was prescribed antipyretics. Over the following week, her symptoms gradually worsened, with increasing cough, shortness of breath, and persistent fever prompting her to come to the emergency department. She was hospitalized and diagnosed with pneumonia. Two nasopharyngeal swabs were taken, and the patient was admitted to a negatively pressurized room with contact, droplet, and airborne transmission precaution protocols in place.

**Fig. 1:**
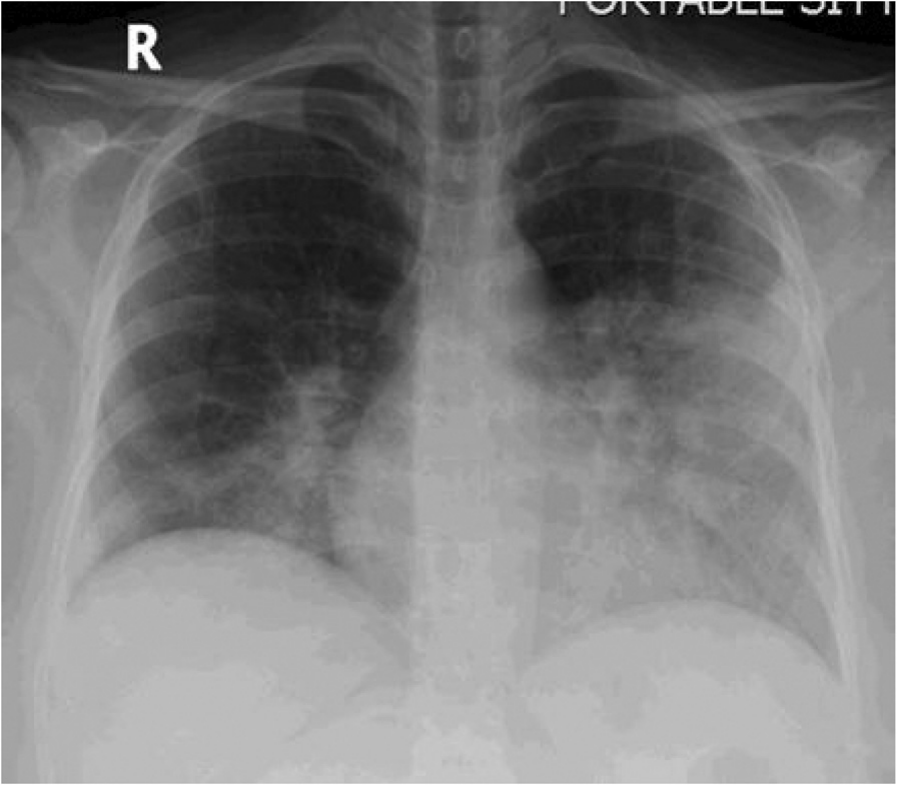
Chest Radiography (postero-anterior view) showing bilateral infiltration

On admission, her highest recorded temperature was 39.2 °C, and her oxygen saturation was maintained via room air (97 − 99 %). Chest radiography showed diffuse bilateral lower lobe infiltrates (Fig. 1). Other laboratory findings included a leukocyte count of 6600/mm^3^ comprised of 81 % polymorphonuclear cells, 13 % lymphocytes, and 5 % monocytes; a platelet count of 127,000/mm^3^; alanine aminotransferase concentration of 254 U/L; and aspartate aminotransferase concentration of 258 U/L. The patient displayed normal renal function. A fetal ultrasound, performed upon admission, demonstrated a live intrauterine fetus of approximately 31 weeks of gestational age, and a posteriorly located placenta. MERS-CoV RNA was not detected in a nasal swab taken at that time.

Treatment with intravenous imipenem, vancomycin, azithromycin and oseltamivir were initiated. Despite antibiotic therapy, the patient became increasingly dyspneic over the following 3 days. Arterial blood gas analysis showed pH 7.34, PaCO_2_ 36 mmHg, and PaO_2_ 73 mmHg while on room air. She also exhibited radiographic evidence of progressive pulmonary infiltrates. Five hours later, the patient developed respiratory failure. She required mechanical ventilation, and dexamethasone was administered twice as prophylaxis for the fetus. A cesarean section was performed at 32 weeks of gestation, and a healthy 1.79 kg preterm male infant was delivered without complications. Following an uneventful delivery, the patient was transferred to the intensive care unit (ICU). Her oxygen requirement increased to 100 %, chest radiography revealed bilateral consolidation, and her condition progressed to acute respiratory distress syndrome (ARDS). Nine days after the initial onset of infection, polymerase chain reaction (PCR) analysis, using real-time reverse transcription PCR (rRT-PCR) targeting genes E and 1a open reading frame, of tracheal aspirate confirmed the presence of MERS-COV infection [9]. Combination therapy with IFN-alpha-2b and ribavirin were initiated, as was methylprednisolone for ARDS. Subsequent septic screening samples including urine, blood, and sputum cultures were all negative for bacterial super infections.

Over the next 4 days, the patient began to improve. Chest radiography showed interval improvement, her fever resolved, her oxygen requirement decreased. Repeated blood tests showed normal leukocyte and platelet counts, and liver function test came back to normal. Five days later, the patient was weaned from mechanical ventilation, and transferred to the medical ward. She continued to recover uneventfully, and was discharged 28 days after hospital admission.

All health care workers taking care of her were asymptomatic with negative nasopharyngeal swab for MERS-Cov PCR, except for a 50-year old female nurse, with no comorbidities, from the obstetrics and gynecology ward. The patient was under her care prior to being transferred to the ICU with progressive and severe pneumonia. She had unprotected exposure for 2 min with 50 cm distance separation between her and the patient. The nurse clearly mentioned that the patient was coughing in her direction during that transient exposure. She was later admitted for 3 days with self-limiting MERS-Cov pneumonia, based on positive MERS-Cov PCR nasopharyngeal swab and bilateral lung infiltrate. She was discharged and sent home in a stable condition.

The male infant in the case reported herein was delivered by emergency cesarean under contact, droplet, and airborne transmission precautions. He was kept in the neonatal unit for observation, and was fed artificial formula in replacement of breast milk. Repeated nasopharyngeal swabs PCR analyses showed a consistently negative result for MERS-CoV. All healthcare workers in contact with the infant during his delivery, and subsequent care, have remained asymptomatic.

## Discussion

The severity of viral pneumonia in pregnancy is evidently related to physiological and immunological changes that result in a shift from cell-mediated to humoral-mediated immunity [10]. Different outcomes in pregnancy have been reported in association with different viral respiratory illnesses, including pandemic influenza, H1N1, and SARS [4]. Among the 12 gravid women who presented with SARS in Hong Kong, between February 1st and July 31st in 2003, 50 % required ICU admission, 33 % required mechanical ventilation, 57 % who presented during the first trimester had spontaneous miscarriages, and 80 % of those presenting late in pregnancy underwent preterm deliveries. Over 80 % of the women gave birth via emergency cesarean secondary to failure at maintain adequate blood oxygen saturation, despite being on 100 % oxygen [4].

In the present report, we have described a serious case of MERS-CoV during the 3rd trimester of pregnancy, requiring mechanical ventilation. This case differs from those reported previously that were associated with stillbirth in the 2nd trimester, during the MERS-CoV outbreak that occurred in Jordan from February through April in 2012 [8]. Our patient had an uneventful perinatal course, and a successful outcome. Various factors may have contributed to this, including the timing of MERS-CoV exposure during pregnancy, her young age, the use of steroids, and potential differences in immune responses. While combination therapy with ribavirin and interferon was administered, it is unlikely that either contributed to the successful outcome; because both were given after delivery, as MERS-COV was not confirmed until later in the course of her illness.

There is limited information available on pregnancy, during antenatal, birth, or postnatal period, and MERS-CoV infection. Antiviral therapy has not yet been approved for the treatment of gravid patients with MERS-CoV infection. A retrospective cohort study in patients with severe MERS-CoV infection showed that the combined administration of ribavirin and IFN-alpha-2a seemed to significantly improve survival rate at day 14 but not day 28 [11]. However, other retrospective studies have failed to show any improvement in the mortality rate following this combination therapy [12]. During the SARS outbreak of 2003, ribavirin was used in pregnant women with the most severe forms of the illness. However, ribavirin therapy increases the risk of teratogenic effects in newborns [13]. Therefore, the use of this drug is not recommended during pregnancy or lactation. Drug treatment of MERS-CoV in pregnancy requires further investigation in the clinical setting.

This report provides an initial view of the outcome associated with pregnancy-related MERS CoV infection. Further data on larger numbers of gravid women infected with MERS-CoV will facilitate a better understanding of the impact of MERS-CoV infection on perinatal outcome.

## Conclusion

Middle East Respiratory Syndrome coronavirus (MERS-CoV) known to cause severe acute respiratory illness associated with a high risk of mortality Various factors may have contributed to the successful outcome of our patient infected with MERS-COV, including young age, presentation in late trimester, and differences in immune responses.

### Consent

Written informed consent was obtained from the patient for publication of this case report and any accompanying images. A copy of the written consent is available for review by the Editor of this journal.

## Abbreviations

ARDS: acute respiratory distress syndrome
H1N1: Hemagglutinin Type 1 and Neuraminidase Type 1 (influenza strain swine flu)
ICU: intensive care units
MERS-CoV: Middle East Respiratory Syndrome coronavirus
PaCO_2_: partial pressure of carbon dioxide in arterial blood
PaO_2_: partial pressure of oxygen in arterial blood
PCR: polymerase chain reaction
SARS: severe acute respiratory syndrome
